# Perspective: Water and Electrolytes in Daily Hydration Compared with Acute Rehydration: Expert Consensus and Research Priorities

**DOI:** 10.1016/j.cdnut.2026.109549

**Published:** 2026-09-29

**Authors:** Maha Tahiri, Paula R Trumbo, Leslie J Bonci, Douglas J Casa, Adam Drewnowski, Jack A Gilbert, Richard J Johnson, Colleen X Munoz, Stavros Kavouras

**Affiliations:** 1 S2B Group, Nutrition Sustainability Strategies, St Petersburg, FL, United States; 2 Paula R. Trumbo Consulting, Charleston, SC, United States; 3 Department of Public Health, School of Health Sciences, Liberty University, Lynchburg, VA, United States; 4 Active Eating Advice, Pittsburgh, PA, United States; 5 Department of Kinesiology, University of Connecticut, Storrs, CT, United States; 6 Department of Epidemiology, Center for Public Health Nutrition, University of Washington, Seattle, WA, United States; 7 Department of Pediatrics, University of California San Diego, San Diego, CA, United States; 8 Center for Marine Biotechnology and Biomedicine, Scripps Institution of Oceanography, University of California San Diego, San Diego, CA, United States; 9 Department of Medicine, University of Colorado, Denver, CO, United States; 10 Department of Health Sciences, Hydration Health Center, University of Hartford, West Hartford, CT, United States; 11 School of Human Performance, College of Health Solutions, Arizona State University, Phoenix, AZ, United States

**Keywords:** hydration, water, electrolytes, chronic disease, biomarkers

## Abstract

Adequate hydration is essential for the normal functioning of the body and health. Adequate hydration is affected by the diet, environmental conditions, social norms, personal preferences, cultural traditions, medical issues, and physical activity. Much research has been conducted on how to best measure various states of hydration, as well as the physiology underlying the regulation of osmolality and hydration. The most current evidence suggests that there is no single reference standard for measuring hydration status, and the most reliable biomarker can vary across situations. Much research has been conducted to understand the physiological processes that regulate hydration (e.g., vasopressin), as well as the associations between hydration, diet, and health. Much of this research has been conducted in animals or as observational studies. There is a need for clinical trials to evaluate causal relationships among diet and lifestyle, physiological regulation, hydration status, and health outcomes. On the basis of the most current evidence, consensus statements, action items, and future research needs were developed.

## Introduction

Water is the largest constituent in the human body, accounting for 50% to 75% of body weight. Water is essential for cellular and metabolic processes. Water in the body exists as an aqueous solution of electrolytes and is present in both the intracellular and extracellular compartments [1,2]. Sodium is the predominant extracellular electrolyte in the extracellular compartment, whereas potassium is the major intracellular electrolyte [1,2]. Chloride is the principal anion in both compartments [1,2]. Magnesium regulates hydration at the cellular level by fueling the “sodium-potassium pump” (using ATP) to maintain the proper balance of water and minerals [1,2]. Phosphate supports cellular hydration by maintaining structural integrity through ATP, which fuels the ion pumps that manage water movement [1,2].

Commonly, hydration status refers to changes in water balance and is reflected by changes in serum osmolality, with low serum osmolality reflecting a gain in water and a high serum osmolality reflecting a loss of water, and this is paralleled by an increase or decrease in intracellular volume, respectively. In contrast, the extracellular fluid space, which consists of plasma and interstitial fluids, can change even without changes in serum osmolality, and this is typically reflected by a gain or loss of sodium. Sweating, for example, results in a loss of both water and salt and can lead to both dehydration (increased osmolality) and extracellular volume contraction from sodium losses. In this paper, we primarily discuss changes in water balance.

Body water balance depends on water net gains (water, beverages, food, and metabolism) and losses (urine, respiration, bowel movements, and skin). Vasopressin is an antidiuretic hormone released in response to increased blood sodium concentrations or low blood volume, which in turn increases water retention by the kidneys, thereby diluting sodium concentrations in the blood and reducing urine output. Increased sodium intake is accompanied by a transient increase in blood sodium and osmolality, which triggers thirst and vasopressin release, resulting in increased water intake and renal water retention; through these mechanisms, serum osmolality is restored toward baseline.

There has been much research in the area of hydration, including validation of existing hydration status biomarkers, identification of new hydration status biomarkers, dietary factors that affect hydration, and the role of hydration in health. This paper presents the perspectives of an Expert Panel of scientists convened by Ultima Health Products Inc. to evaluate the current state of the science regarding the physiological regulation, assessment, and health effects of hydration. In addition, the Expert Panel was charged with identifying consensus statements and priorities for future research and action in this area.

## States of Hydration

There are various terms used to describe the body’s losses and gains of water and the state of hydration. Individuals are either in a state of dehydration/hypohydration (condition of body water deficit), euhydration (condition of normal body water), or hyperhydration (condition of body water excess) [3]. Rehydration is the process by which adding water shifts the state of hydration from dehydration to euhydration, and overhydration shifts the state from euhydration to hyperhydration. According to the National Library of Medicine of the United States, dehydration is the condition that results from excessive loss of water from a living organism [4]. Hydration status is a continuum, and even within the state of dehydration, there can be various states (e.g., minimally dehydrated, significantly dehydrated, and seriously dehydrated). Precise, consensus-backed definitions of hydration state, however, are absent from the scientific literature and are needed for consistency in clinical practice, regulatory claim language, consumer education, and future research design.

## Assessment of Hydration Status

There are numerous methods and biomarkers used to measure hydration status, and there is no particular reference standard (Table 1) [[5], [6], [7], [8], [9], [10], [11], [12], [13], [14], [15], [16], [17], [18]]. Several studies and reviews have been conducted to validate and evaluate the reliability of these methods. In one study, various hydration assessment markers were measured in individuals with high and low water intake [5]. It was shown that 24-h urine osmolality, 24-h urine volume, and saliva osmolality were sensitive measures of habitual differences in total water intake, as well as changes in total water intake. In another study, urine concentration (osmolality, specific gravity, and color) and volume responded to changes in water intake and stabilized within 24 h of change [6]. At rest, it was shown that only the urinary indices increased in accordance with body water loss [7]. During exercise, however, serum osmolality, salivary osmolality, and urine osmolality exhibited the highest sensitivity. Urine volume at 60 min after consuming a water bolus was inversely correlated with the entering hydration state when assessed by urine specific gravity and urine osmolality. Using urine osmolality as a reference, it was reported that urine color alone and in combination with urine void number is a valid, inexpensive field measure to detect simple underhydration [8].

**TABLE 1:** Biomarkers and methods to measure hydration status, organized by use context

Biomarker or method	What it reflects	Setting	Key considerations
Acute fluid loss and rehydration (minutes to hours; exercise, heat exposure, acute illness)			
Plasma osmolality	Extracellular tonicity; the regulated variable of body water balance	Clinical	Considered the best single marker for diagnosing acute dehydration and among the most sensitive measures during exercise [7,12]. Requires venipuncture and osmometry. Values fall rapidly after drinking, before body water stores are fully restored.
Plasma volume change	Relative change in intravascular volume, from hemoglobin and hematocrit	Research laboratory	Sensitive to acute intravascular shifts during exercise and heat exposure, but reflects redistribution among fluid compartments rather than a change in total body water. Baseline values are altered by posture and by prior exercise.
Blood volume	Absolute intravascular volume	Research laboratory	Requires indicator dilution or carbon monoxide rebreathing. Accurate but impractical outside specialized laboratories, and expanded by heat acclimatization and endurance training independently of hydration status.
Total plasma protein	Hemoconcentration accompanying loss of plasma volume	Clinical	An inexpensive surrogate for plasma volume change. Insensitive to small fluid deficits and confounded by posture, protein-energy status, inflammation, and liver or kidney disease.
Saliva osmolality	Salivary gland response to changes in body water and tonicity	Field; laboratory	Among the most sensitive markers during exercise [7] and noninvasive to collect, but strongly affected by salivary flow rate, mouth breathing, and recent food or fluid intake. Requires a standardized collection protocol.
Sweat composition	Rate and electrolyte content of sweat losses	Field; laboratory	Quantifies the fluid and sodium losses that must be replaced rather than hydration status itself and supports individualized fluid replacement during exercise [10]. Varies widely between individuals and with sweat rate, heat acclimatization, and collection site. Wearable sweat sensors are advancing, although validation for real-time assessment of hydration status is ongoing.
Chronic hydration status (days to weeks; free-living conditions and habitual water intake)			
Copeptin	Vasopressin secretion, and therefore the neuroendocrine burden of low water intake	Clinical; research	Outperforms urine osmolality and 24-h urine volume as a marker of chronic hydration status because it reflects the neuroendocrine burden of underhydration rather than the renal response alone [13]. Requires venipuncture, and assay cost and limited availability currently restrict use to research and specialized clinical settings.
Serum sodium	Extracellular tonicity, integrating habitual water balance over time	Clinical	Stable enough for day-to-day monitoring and a powerful population-level marker of chronic underhydration in observational studies [14]. Risk is predicted above 142 mmol/L, at the upper end of the normal range of 135–145 mmol/L, so high-precision measurement is needed to separate risk categories [14,15].
Urine volume (24-h)	Renal water excretion across a full day	Field; clinical	Sensitive to changes in total water intake and stabilizes within ∼24 h of a change [5,6]. Requires a complete 24-h collection, which limits compliance in free-living settings. Cumulative urine output after a standardized water load is used separately to compare beverages (beverage hydration index) [18].
Total body water	Absolute quantity of water in the body	Research laboratory	Typically determined by isotope dilution and used as the criterion measure of body water. A single measurement describes body water content rather than water balance, and interpretation requires normative values for age, sex, and body composition. Stability over weeks in free-living adults has been questioned [16].
Multiple-frequency bioelectrical impedance	Total body water and its distribution between the intracellular and extracellular compartments	Clinical; research	Portable and noninvasive, but estimates depend on population-specific prediction equations and are sensitive to recent food and fluid intake, exercise, posture, and skin temperature. Not sufficiently precise to detect small acute fluid changes.
Neutron activation analysis	Elemental body composition: a criterion method for body compartments	Research laboratory	Highly accurate, but requires specialized facilities and involves radiation exposure, restricting its use to a small number of research centers.
Urge to urinate and void frequency	Perceived bladder filling and daily voiding pattern	Field	Requires no equipment, which suits it to self-monitoring and public health messaging. Void number combined with urine color is a valid and inexpensive field indicator of simple underhydration [8]. Confounded by bladder capacity, caffeine and alcohol, diuretics and other medications, and urological conditions.
Applicable in both contexts, with context-dependent interpretation.			
Body weight	Net change in total body water when energy balance and body composition are stable	Field	Acute: the practical criterion for fluid loss and replacement during exercise, expressed as percent body weight loss, with thirst engaged near 1%–2% loss [9]. Chronic: serial first-morning body weight is one of the 3 WUT criteria [11], although over days to weeks, changes in energy balance and body composition confound interpretation.
Urine osmolality	Renal concentrating response to vasopressin	Field; clinical	Acute: among the most sensitive markers during exercise [7]. Chronic: 24-h urine osmolality is sensitive to total water intake and stabilizes within ∼24 h of a change [5,6]. Spot samples vary with circadian rhythm and time since the last drink, and reflect the renal response rather than the neuroendocrine burden of underhydration [13].
Urine specific gravity	Urine density, reflecting total solute concentration	Field	Inexpensive and rapid by refractometer, and widely used both for preexercise screening and for day-to-day monitoring. Tracks urine osmolality closely but is disproportionately affected by large solutes such as glucose, protein, and contrast media. Like all spot urine indices, it lags acute fluid intake.
Urine conductivity	Ionic (electrolyte) concentration of urine	Field	A rapid, low-cost surrogate for urine concentration using a handheld device. Because it detects only ionic solutes, it is influenced by dietary electrolyte load independently of water balance, and it is less extensively validated than urine osmolality or specific gravity.
Urine color	Urochrome concentration, reflecting urine concentration	Field	The most accessible field measure and one of the 3 WUT criteria [11]. Validated against urine osmolality for detecting simple underhydration, both alone and in combination with void number [8]. Confounded by B vitamins and other supplements, by some foods and medications, and by lighting conditions.
Thirst (in combination with other markers)	Perceived drive to drink, triggered by rising plasma osmolality and falling blood volume	Field	Engaged at ∼1%–2% body weight loss in young adults but blunted in older adults [9], and unreliable after exercise because drinking can restore body weight without restoring osmolality [9,10]. Most informative as one of the 3 WUT criteria rather than on its own [10,11].
Skin moisture	Water content of the superficial skin layers	Field	Noninvasive and quick, but influenced by ambient temperature and humidity, measurement site, and skin care products. Validation against reference measures of body water is limited, and it is not recommended as a stand-alone indicator in either context.

Abbreviation: WUT, body weight, urine color, and thirst.

Biomarkers and methods are grouped by the use context in which they are most informative. No single measure is a reference standard: one blood or urine sample cannot validly represent fluctuating total body water and fluid compartments, and combining markers increases accuracy [17]. Selection should therefore be guided by the time course of the fluid challenge (minutes to hours compared with days to weeks), the setting and resources available, and the population being assessed. Setting: field, suitable for self-monitoring or on-site use with minimal equipment; clinical, requires venipuncture or routine clinical laboratory analysis; research laboratory, requires specialized equipment, facilities, or expertise.

Armstrong et al. [9] evaluated the validity and efficacy of thirst sensation and urine volume in individuals with different hydration states. Thirst activates at ∼1% to 2% body mass loss in young adults but is blunted in older adults. Thirst is unreliable postexercise because postexercise rehydration can restore body weight without restoring osmolality. Thirst was positively correlated with body water loss and distinguished dehydrated individuals from euhydrated individuals. The limitations of thirst were also reported by Adams et al. [10].

The combination of body weight, urine color, and thirst (WUT) is commonly used to measure hydration status. One study reported that when all 3 criteria based on WUT markers are met for dehydration, urine specific gravity and urine osmolality indicate dehydration, and when none of the WUT dehydration markers are met, there is a high likelihood of euhydration [11]. This finding is further supported by Adams et al. [10], who concluded that changes in body mass and thirst perception were the most influential predictors of dehydration.

Plasma osmolality is considered the best biomarker for diagnosing acute dehydration. Values > 285 mmol/kg consistently predict elevated plasma copeptin [12]. Using plasma arginine vasopressin (AVP) concentration to analyze multiple published datasets, it was determined that a mild neuroendocrine defense of body water begins when total water intake is < 1.8 L/24h [19]. Copeptin, a surrogate biomarker of vasopressin, outperforms urine osmolality and 24-h urine volume as a marker of chronic hydration status because it reflects the neuroendocrine burden of underhydration, not merely renal response [13].

Serum sodium at the population level is a powerful tool in observational studies for identifying chronic underhydration and a stable marker for day-to-day monitoring [14]. The range that predicts risk (> 142 mmol/L) is at the higher end of the normal physiological range (135–145 mmol/L), therefore requiring high-precision measurement to distinguish risk from high risk [14,15].

On the basis of short-term studies, it has been thought that total body water is stable over time in healthy individuals under daily life conditions, regardless of the volume of water consumed, but this may not be the case. One small (*n* = 5) longer-term study showed that indices of body water volume did not remain stable over time [16]. Over 4 wk, body weight significantly increased and saliva osmolality significantly decreased, despite no significant changes during the study period in environmental conditions, total energy or macronutrient intake, or time spent doing moderate or vigorous physical activity. Serum osmolality also decreased significantly.

The above are a few examples of the many studies conducted on measures of hydration status. It has been concluded from these studies that *1*) hydration markers should be selected by considering daily activities; *2*) one blood or urine sample cannot validly represent fluctuating total body water and fluid compartments [17], and rather, combinations of hydration markers increase accuracy because of context/practical limitations of each method; and *3*) assessment choice depends heavily on practical and situational factors (e.g., availability, feasibility, acute compared with chronic status, environmental conditions).

Although this paper examines the best options for the general population to examine hydration status on a daily basis that will be easy to do, adequately valid, and inexpensive, it is important to note that the advancement of technology in the wearable space related to hydration status may shift how hydration status is monitored in the near future. The potential upside of real-time assessment that is fed through an app on the phone or watch will allow for more consistent feedback of fluxes in fluid levels. This could be especially valuable in circumstances where medical conditions/medications alter fluid regulation, for individuals who do extensive exercise/labor in the heat for many hours a day, and for those who find it cumbersome to use traditional metrics for hydration status tracking. Although these wearables have made considerable progress related to sweat volume and sweat electrolyte concentrations, we are still in the process of establishing the validity of wearable products that can establish an accurate assessment of real-time hydration status.

## Fluids, Electrolytes, and Hydration

There are 2 general ways to evaluate the effect of fluids and electrolytes on hydration. The beverage hydration index (BHI) is an acute measure of the relative hydration properties of different beverages, using water as a standard. In essence, it measures the relative retention of fluid to plain water. Subjects consume 1 L of water or test beverages, and urine is collected for 4 h. BHI is calculated as the ratio of the cumulative urine volume for deionized water to the cumulative urine mass of the test beverage at specific time points (e.g., hourly ≤240 min) after ingestion [18]. In the first study using the BHI, it was demonstrated, using 13 different beverages, the drinks with the naturally highest electrolyte content (e.g., orange juice and milk) tended to have the highest BHI [18]. It was further demonstrated that electrolytes (i.e., sodium and potassium) make the biggest contribution to hydration when compared with carbohydrates and protein [20]. Although the results of the BHI studies are based on the acute effects of a large bolus of fluid over the subsequent 4 h, there is evidence that the results may be extrapolated to a longer time scale [21]. A limitation of this test is that it does not address changes in osmolality that reflect true hydration state but rather reflect a general measurement of fluid retention.

Exercise-induced dehydration followed by rehydration is another approach for evaluating the acute effect of fluids and electrolytes on hydration. This protocol involves 2 to 3 h of low-intensity exercise in the heat to induce a controlled 2% to 3% dehydration. This is followed by a 100% to 150% rehydration of the fluids (urine and sweat) lost during 1 h. The primary outcome is to determine how closely body weight returns to baseline over the subsequent 3 to 4 h. Shirreffs et al. [22] varied the percent of rehydration (50%, 100%, 150%, and 200%), as well as sodium content, and concluded that a drink volume greater than sweat loss during exercise must be ingested to restore fluid balance. Sodium content of the beverage, however, must be sufficiently high, or else rehydration will merely result in increased urinary output. One study examined the effect of the sodium content of drinks on rehydration after exercise-induced dehydration equivalent to 1.9% of body mass of euhydrated men [23]. After dehydration, they consumed drinks with varying sodium concentrations. The volume of urine produced was influenced by the amount of sodium consumed, with the greatest volume after ingestion of a 2 mmol/L drink and the least after a 100 mmol/L drink. The importance of sodium content was also reported by Wemple et al. [24]. This likely reflects the need to replace not only water but salt losses from sweating induced by exercise.

There is no published clinical trial that has directly manipulated sodium, potassium, and magnesium, or their ratios, in a daily hydration context among free-living nonathletic adults and measured 24-h fluid balance. As such, very little is known about potassium, calcium, and magnesium’s contribution to hydration efficacy in any context, and the effect on hydration status when the electrolyte ratios are manipulated. It is also necessary to draw a distinction between the nutrient profiles of beverages suited for daily hydration compared with recovery from excessive fluid loss (which may require greater sodium and potential carbohydrates).

Studies have shown that other food components can affect thirst and/or hydration. Flavored beverages are manufactured to help encourage children to drink fluids. A flavored, low-sugar beverage enhanced ad libitum fluid intake and improved hydration markers compared with water during exercise in the heat, supporting its potential as a practical rehydration strategy for children [25]. In contrast, experimental studies suggest that soft drinks do not hydrate but rather increase serum osmolality and may even induce kidney damage in heat-stressed, dehydrated rats [26]; clinical studies also suggest that soft drinks may increase the risk for kidney injury in heat-stressed environments [27]. Milk, which contains sodium, can also be an effective postexercise rehydration drink [28]. A hypertonic milk permeate-based sports drink was reported to be more effective for postexercise rehydration (plasma volume restoration) than traditional sports drinks, high-potassium sports drinks, and water when consumed at 150% of body weight lost [29]. When provided in a volume equivalent to 125% of exercise-induced fluid loss, an amino acid oral rehydration solution produced comparable/superior fluid balance and superior sodium/chloride balance responses to popular glucose-based and sugar-free oral rehydration solutions [30]. Individualized fluid replacement strategies are optimal for minimizing the extent of fluid losses during exercise for optimal performance [10].

Continuous passive water losses through urine, respiration, and skin are the dominant fluid challenge facing the general population under ordinary conditions, and these losses are best replaced by net fluid intake that is primarily hypotonic and water-dominant. This stands in contrast to the higher sodium and carbohydrate compositions that are appropriate and well-supported by exercise science for acute rehydration after significant fluid loss, such as that occurring with 2% or greater body mass loss during exercise or other high-output physical activity. Conflating these 2 contexts has practical consequences. Mean American sodium intake already exceeds the adequate intake (AI) by ∼2-fold, and routine consumption of high-sodium beverages framed as hydration products risks compounding an existing public health burden rather than addressing a physiological need. Daily hydration beverages are therefore best evaluated through a nutrient density lens, delivering minerals and electrolytes associated with fluid balance, including potassium, magnesium, and calcium, without contributing to the documented dietary excesses of sodium and added sugars. Inadequate intake of magnesium is common in the United States, with almost half (48%) of the United States population consuming less than the recommended amount of magnesium from food in 2005–2006 [31]. Serum magnesium concentrations (a marker of magnesium status) are inversely correlated with the degree of obesity [32]. Only 53% of Americans aged ≥2 y consume the daily value of 4700 mg/d potassium [33]. Recognizing hypotonicity as a key principle underlying daily hydration, distinct from the formulation requirements of acute rehydration, is essential for advancing scientific clarity, regulatory coherence, consumer understanding, and product development in this category.

## Dietary Reference Intakes for Total Water

The Institute of Medicine set Dietary Reference Intakes for total water (pure water, beverages, and food) based on median intake data from NHANES III (1988-1994) [34]. NHANES began systematically collecting data on all types of water intake (tap, bottled, plain, or carbonated) as part of the 24-h dietary recall in the 2005–2006 survey cycle. Before this, water intake was evaluated using postrecall food-frequency questionnaires rather than the detailed 24-h dietary recall. It was determined that median total water intakes for each age and gender group met the definition of an AI because these intake concentrations were associated with normal serum osmolality (NHANES 1988–1994), an indicator of hydration status [35]. As such, on the basis of this particular survey, the AI for total water is intended to be adequate for 50% of the population. The AI is not intended to assess the inadequacy of a nutrient for populations [34]. The AI for total water intake is 1.3 to 3.3 L/d for children and adolescents, and 2.7 and 3.7 L/d for women and men, respectively [34].

## US Water Intakes

Although NHANES III (1988–1994) provided estimates of daily water intake, the current detailed, standardized 24-h dietary recall NHANES for all sources of water began with the 2005–2006 cycle. On the basis of NHANES, the primary contributors of water were pure water (39%), followed by beverages (38%), and food (23%) [36,37]. Food moisture content varies among foods, with content being high in fruits and vegetables and low in grains [38,39]. On the basis of more recent NHANES 2011–2016 data, the percentage of Americans not meeting the AI for total water was ≥70% for children and adults [40]. Older men and adolescents had the highest percentage of not consuming the AI. In the 18 to 24 y age group, less than half of all young adults and only 18.7% of non-Hispanic Black young adults met the AI for water [41]

Average consumption of sugar-sweetened beverages has declined from ∼322 mL/d (2011–2012) to 262 mL/d (2015–2016), whereas water intake has increased from 1011 mL/d (2011–2012) to 1144 mL/d (2015–2016) [40]. Sodium and potassium intakes generally remained unchanged during 2011–2020, with a sodium:potassium ratio of 1.4. There are cultural differences in the source of water consumed, with the majority of Caucasian Americans consuming tap water and Hispanic Americans consuming bottled water [40]. Consumption of certain beverages is more prevalent at certain times of the day than others. For example, water, coffee, and tea are most commonly consumed by adults in the morning, whereas alcohol is consumed at night. Dietary pattern quality (e.g., Healthy Eating Index) correlates positively with water intake, suggesting hydration inadequacy clusters with other nutritional factors [40]. Populations with lower diet quality and socioeconomic status tend to consume less water and have higher consumption of sugar-containing beverages.

Analysis of water intake in the NHANES datasets predates 2019, meaning that NHANES lags the market, and many new beverage subcategories (e.g., electrolyte beverages, enhanced beverages, sports/energy drinks, and functional hydration products) and point-of-sale products (e.g., e-commerce) are not captured in the older data. There is a need to recategorize beverage groups in line with current market offerings and nutrient database (Food and Nutrient Database for Dietary Studies) definitions, and confirm that NHANES and national food composition databases fully capture the new beverage subcategories. Once this is completed, an updated NHANES analysis (2018–2023) on water intake can be conducted to capture the emerging beverage subcategories. Such information can be used to better understand the association between certain types of beverages and osmolality and health.

## Hydration, Copeptin, and Body Weight

Copeptin is a glycopeptide derived from the same precursor as AVP and is released into the circulation in equimolar concentrations with AVP (i.e., 1 mmol of AVP is secreted with 1 mmol of copeptin). As a result, copeptin is considered a reliable and clinically useful surrogate marker of AVP secretion. Compared with AVP, copeptin is a larger molecule, has a longer half-life, is highly stable in vitro and ex vivo, and can be measured more easily and accurately using immunoassays [42]. Copeptin is released when plasma osmolality exceeds ∼285 mmol/kg to regulate body water and blood pressure. Vasopressin also functions as a fat-storage signal to help animals survive food and water scarcity by promoting energy storage as fat and glycogen [43].

In a Swedish population-based sample, elevated plasma copeptin was found to be associated with incident diabetes mellitus independent of confounders, including plasma glucose and insulin [42]. Furthermore, in the same population, elevated plasma copeptin was found to be cross-sectionally associated with metabolic syndrome, obesity, hypertension, abdominal obesity, and high C-reactive protein. On the basis of an analysis of NHANES 2009–2012 data, underhydration was found to be associated with obesity and other chronic diseases in the United States population aged 51 to 70 y [44]. To investigate the association between serum sodium concentrations and obesity, it was shown that when salt was added to the diets of mice, they gained more weight and had more visceral fat compared with those fed a control diet [45]. In addition to salt, fructose, which is a component of sucrose in sugar-sweetened beverages and raises serum osmolality, can also cause obesity and metabolic syndrome [43]. Mice that cannot metabolize fructose, for example, are protected from developing fructose- or salt-induced metabolic syndrome [45,46]. Hydration was shown to reverse fructose–induced obesity in these mice. Lanaspa et al. [45] also showed that high salt intake activates the aldose reductase, fructokinase pathway in the liver and hypothalamus, leading to endogenous fructose production with the development of leptin resistance and hyperphagia that can cause obesity, insulin resistance, and a fatty liver.

Although the evidence is limited in humans, one study showed that 1 wk of increased water intake led to a significant 15% reduction in copeptin compared with the control, in association with a reduction in fasting plasma glucose [47]. The greatest reduction occurred among subjects with habitually high copeptin and concentrated urine (“water responders”). Analysis of the Korea National Health and Nutrition Examination Survey 2008–2010 showed that higher urine specific gravity was associated with increased trunk fat and lower leg fat [48]. Approximately 19.3% of the effect of urine specific gravity on fat distribution was mediated via insulin resistance, and 13.0% of the effect on insulin resistance was mediated via poor fat distribution. As such, on the basis of observational studies, increased urine specific gravity, a sign of low hydration status and presumably high vasopressin, was associated with insulin resistance and poor fat distribution.

## Hydration and Metabolic Health Indicators

Elevated plasma copeptin is associated with multiple indicators of metabolic syndrome [49]. A Swedish prospective cohort showed that increasing water intake was associated with low triglycerides and high HDL cholesterol concentrations [50]. Furthermore, increased urine osmolality was associated with high plasma glucose and closely associated with high hemoglobin A1c. Plasma copeptin concentration negatively correlated significantly with water intake and urine osmolality. High copeptin concentrations were associated with all the metabolic indicators measured. On the basis of observational studies, these results suggest that low concentrations of copeptin are associated with high water intake, low urine osmolality, and a favorable metabolic profile.

## Hydration and Risk of Chronic Disease and Mortality

A review of the literature was conducted to evaluate the level of evidence for low water intake and risk of various health endpoints [51]. The level of water intake was determined either by measuring water or beverage intake or by measuring biomarkers of dehydration. Numerous observational studies have been conducted on the association between underhydration and risk of kidney stones and type 2 diabetes, with the majority showing a significant association. The findings on the association between hydration and other disease endpoints, including certain cancers, kidney disease, and mortality, are mixed, with the evidence primarily or exclusively based on observational studies. Lacking are clinical trials that are capable of evaluating a causal relationship.

Because the most common cause of increased blood sodium is decreased body water, studies have also evaluated the association between sodium intake/serum sodium and other relevant factors and risk of chronic diseases and mortality. A 5-y cohort study showed that elevated serum sodium was an independent risk factor for the development of chronic kidney disease with an 18% increased risk for every 5 mmol/L change in serum sodium [52]. Calculated serum osmolality was also an independent risk factor for chronic kidney disease. Other studies have linked high salt intake with increased risk of fatty liver, diabetes, and obesity [45,53]. A prospective cohort observed that middle-aged adults with a serum sodium of 135 to 146 mmol/L predicted faster biological aging and greater chronic disease burden (e.g., heart failure, dementia, chronic lung disease, and stroke) [14]. A clinical threshold was determined to be 142 mmol/L with vasopressin release stimulated at 140 to 142 mmol/L, representing 285 mmol/kg.

## Hydration, Mood, Cognition, and Other Functional Effects

A number of trials have been conducted to evaluate the effect of hydration status on mood, cognition, and fatigue. From these trials, several meta-analyses have been conducted [[54], [55], [56]]. These meta-analyses collectively concluded that *1*) when >2% of body weight was lost because of dehydration, there was a significant effect on mood, alertness, and fatigue; *2*) the affected cognition domains that were affected included attention, executive function, and motor coordination; *3*) cognitive performance declined along with the magnitude of water deficit; and *4*) it was challenging in quantifying the effects due to lack of standardized approaches to assessing cognition and hydration status. Conclusions of a narrative review by Liska et al. [57] were that *1*) despite variability among study methodologies, dehydration impairs cognitive performance for tasks involving attention, executive function, and motor coordination when water deficits exceed 2% body mass loss; *2*) cognitive domains involving lower-order mental processing (e.g., simple reaction time) are less sensitive to changes in hydration status; *3*) results from studies on hydration and cognition in children were mixed; and *4*) hypohydration is associated with increased negative emotions such as anger, hostility, confusion, depression, and tension, as well as fatigue and tiredness. These findings are consistent in adults but unclear and very limited in children. Study findings on these functional endpoints since the publication of these meta-analyses and reviews have concluded that, in the elderly, higher dehydration is associated with a steeper decline in cognitive functioning and well-being over time [58], and lower hydration status was associated with greater decline in global cognitive function that included 8 different assessments [59]. In a cross-sectional study, dehydrated individuals more frequently reported fatigue (44%) [60]. Increased water consumption resulted in a significant decrease in fatigue/inertia and confusion/bewilderment and trended toward lower sleepiness compared with baseline [61]. Other potential functional effects that have been reported with changes in hydration status include muscle mass [62], coagulation [63], and oxidative stress [64].

## Hydration and the Gut Microbiota

The gut microbiota is now recognized as a modifiable contributor to cardiometabolic, immune, and neurological disease, and the past 5 y have moved microbiome science meaningfully toward the clinic, with U.S. Food and Drug Administration–approved live biotherapeutics for recurrent *Clostridioides difficile* infection and a growing pipeline of microbiome-informed diagnostics, prognostics, and therapeutics across oncology, gastroenterology, and metabolic disease [65]. Compositional and functional features of stool communities can now stratify patients by adiposity, glycemic status, and cardiometabolic risk, with short-chain fatty acids (SCFAs) emerging as candidate metabolic biomarkers that vary systematically with body composition and geography [66]. Diet is the dominant modifiable input, whereby saturated fat and refined sugar expand facultative anaerobes and disrupt the diurnal microbial oscillations that entrain hepatic clock genes, lipogenesis, and host energy metabolism [67], whereas fermentable fiber promotes SCFA production and a more diverse, resilient community. Given that nutrients consumed in gram quantities exert these effects, it is biologically plausible that water, which is consumed in far greater volumes and the matrix in which dietary chemistry and electrolytes are delivered, also shapes the microbiota.

In mice, restricting water intake to 25% and 50% of normal for 14 d induced constipation without overt dehydration, shifted gut microbial composition, depleted colonic Th17 cells, and impaired clearance of *Citrobacter rodentium* [68]. Although evidence suggests that reduced water intake is associated with increased risk of constipation [69], evidence for the effect on gut microbial composition is lacking. In the American Gut cohort, drinking-water source (tap, filtered, bottled, or well) ranked among the strongest extrinsic determinants of fecal microbiota composition, and intake volume was independently associated with distinct community signatures [70]. In a United States cross-sectional study, no association was observed between objective hydration biomarkers (total water intake, urine specific gravity, and copeptin) and overall microbial community structure, although differential abundance of specific taxa correlated with plasma lipopolysaccharide-binding protein, hinting at links to intestinal barrier integrity [71]. A randomized crossover in healthy men similarly found no differential effect of alkaline compared with neutral-pH water (2 L/d) on overall diversity or community structure [72]. Indirect evidence from electrolyte perturbations is consistent with a mediating role, whereby oral NaCl supplementation in neonates with enterostomies reshaped intestinal communities alongside improved weight gain [73], and pediatric nonalcoholic fatty liver disease (now known as metabolic dysfunction-associated steatotic liver disease) shows coupled disturbances in microbiota composition and water–electrolyte metabolism [74]. Prospective trials that vary water volume and composition (mineral content, electrolytes, and pH) and that pair stool, immune, and metabolite readouts with validated hydration biomarkers are needed to determine which microbial signals are sensitive, specific, and clinically actionable.

## Behavior Change toward Increased Hydration

Fluid intake can be less than desirable for some individuals and for different reasons. Some individuals do not like the taste of water, whereas some only drink when thirsty or dislike eating fruits and vegetables. Other barriers to proper hydration include food/beverage insecurity, medications, medical conditions, increased physical activity, diarrhea/vomiting, weight-loss diets (e.g., keto diets), the need to avoid urinating, hot climates, and limited access to fluids for those who have mobility issues (e.g., the elderly).

Many health professionals, as well as the United States government (e.g., NIH, USDA, and Centers for Disease Control and Prevention) and health organizations (e.g., National Kidney Foundation and Academy of Nutrition and Dietetics) stress the importance of hydration and provide guidance for increasing fluid intake [[75], [76], [77], [78], [79]]. Strategies for increasing water intake include regularly consuming fluids with and between meals and snacks, including fruits and vegetables as part of meals, drinking higher-osmolality drinks, such as milk and juices, avoiding drinks with added sugars that contribute to dehydration, and adding a flavor enhancer for variety. Simple questions to ask oneself for specifically assessing hydration status include the following: *1*) Are you thirsty throughout the day? *2*) Do you feel weak, dizzy, or lightheaded? *3*) Is your mouth dry? *4*) Does your urine appear dark yellow in color? and *5*) Do you have an urge to urinate, and how frequently?

There are many “hydration” products currently on the market, and they vary widely in content. These products also vary in the identification of the product, such as “electrolyte beverage.” These terms are not universally understood by the consumer and vary in formulations, with some containing sugar and varying levels of electrolytes.

## Advancing Public and Scientific Knowledge in Hydration

Hydration has historically received less attention from the nutrition science community than other dietary components, yet the evidence reviewed herein indicates that this imbalance warrants reconsideration. A majority of Americans do not meet the AI for total water, and chronic underhydration has been associated with elevated vasopressin and copeptin, elevated serum sodium, and a range of adverse health outcomes, including obesity, type 2 diabetes, chronic kidney disease, accelerated biological aging, and impaired cognitive function and mood [44]. Although much of this evidence is derived from observational studies and animal models, the consistency of associations across cohorts, populations, and physiological endpoints suggests that adequate daily hydration is not solely a matter of comfort or short-term performance but a modifiable factor with potential relevance to the prevention of chronic disease. The implication is that hydration warrants the same level of scientific investment, policy attention, and public health communication that has been directed toward other dietary components. The nutrition field has an opportunity, and arguably a responsibility, to advance the evidence base through clinical trials, refined assessment tools, and updated dietary guidance that reflect both the benefits of adequate hydration and its role in mitigating risk of chronic disease at the population level.

The Daily Hydration Roundtable came to a consensus on various issues related to hydration (Table 2), as well as identified knowledge gaps requiring action and research (Table 3). It was noted that the scientific literature and other sources of information are not consistent in the terminology used to describe the different levels of hydration status. As such, an immediate action item that was identified was to develop uniform hydration definitions to be used in clinical settings and for regulatory language, consumer education, and future research design and publications.

**TABLE 2:** Consensus statements on hydration

I. Prevalence and public health risk
Underhydration as the primary public health hydration risk
On the basis of dietary intake data from NHANES, a majority of Americans do not meet the adequate intake (AI) for water. Chronic underhydration, distinct from acute dehydration, represents the primary public health hydration risk in the United States population. It is largely silent, compensated by physiological mechanisms, and disproportionately affects older adults and children. Updated prevalence estimates using the most current NHANES cycles are a research priority.
II. Vasopressin, serum sodium, and health outcomes
Elevated vasopressin and metabolic disease associations
Elevated vasopressin, measurable by copeptin, is associated with chronic underhydration and metabolic disease outcomes, including obesity, type 2 diabetes, accelerated biological aging, and cardiovascular and renal disease. These associations are primarily derived from epidemiological studies in humans; causal language is not yet warranted without further mechanistic and interventional evidence.
Elevated serum sodium and chronic disease risk
Elevated serum sodium, within the upper portion of the clinical normal range, is independently associated with adverse health outcomes, including cardiovascular disease, dementia, stroke, diabetes, and chronic lung disease. Although elevated serum sodium may serve as a practical clinical proxy for vasopressin activity and inadequate fluid intake, the direct causal linkage between serum sodium, hydration status, and these disease outcomes requires further investigation.
III. Cognitive and functional impacts of hydration status
Underhydration, dehydration, and cognitive function
Underhydration and dehydration have negative consequences on cognitive function, mood, and fatigue across age groups. Evidence is most robust at or >2% body weight loss, where impairment is reliable and well-documented across multiple cognitive domains. Evidence at lower levels of fluid deficit also exists, although the literature is more varied in its methods and endpoints. This body of evidence is sufficient to substantiate structure/function claims linking adequate hydration to the maintenance of normal cognitive function, mood, and energy levels.
IV. Electrolytes, minerals, and micronutrients
Potassium, magnesium, calcium, and cellular hydration
Potassium, magnesium, and calcium are functionally essential to cellular hydration mechanisms and are consumed in inadequate amounts by a substantial proportion of the United States population, with the greatest shortfalls concentrated among those at the highest cardiometabolic risk. Mineral- and electrolyte-containing beverages represent a practical and evidence-based delivery vehicle, particularly for potassium; evidence for beverage delivery of magnesium and calcium is more limited and warrants further study. Both terms, minerals and electrolytes, should be used together in scientific and consumer communications to bridge physiological and consumer understanding.
Silent inadequacies in mineral intake and chronic disease progression
Inadequate dietary intakes of potassium, magnesium, and calcium may contribute to an asymptomatic, undetected progression toward chronic disease that is not captured by standard clinical measurements, because the body draws on compensatory reserves, including bone, red blood cells, and intracellular stores, before serum concentrations fall below clinical thresholds.
V. Daily hydration and beverage composition
Hypotonic composition for daily hydration
Hydration status is determined by the balance of water and electrolytes, predominantly sodium. For daily maintenance hydration in the general population, net fluid intake should be primarily hypotonic (water-dominant) to compensate for dietary osmotic load and continuous passive water losses. This is distinct from the higher sodium and carbohydrate compositions appropriate during intense exercise and for rehydration after significant fluid losses.
Nutrient density as the standard for daily hydration beverages
Daily hydration beverages should be evaluated through a nutrient density lens, providing minerals and electrolytes associated with fluid balance, including potassium, magnesium, and calcium, without contributing to the public health burden of excess sodium and sugar. The beverages designed for daily hydration represent an opportunity to help bridge documented nutrient gaps rather than compound existing dietary excesses.
Electrolytes in acute rehydration compared with daily hydration
After significant fluid loss, such as that occurring with 2% or greater body mass loss during exercise or other high-output physical activity, the addition of electrolytes, primarily sodium, to replacement fluids supports fluid retention and effective rehydration. This evidence base is relatively robust and derived primarily from exercise-induced dehydration studies in the heat. For routine daily hydration in the general population, evidence from randomized controlled trials is limited, and given that average American sodium intake already exceeds the adequate intake by ∼2-fold, daily hydration should not be assumed to require elevated sodium concentrations. Research priorities should include well-designed trials examining electrolyte needs specifically in the context of daily, nonexercise-related hydration while food is also ingested alongside the tested beverages.
VI. Assessment, biomarkers, and definitions
No reference standard for hydration assessment in free-living populations
No single reference standard exists for assessing hydration status in ambulatory, free-living populations. Accurate assessment benefits from integrating multiple biomarkers, such as body weight, urine characteristics, and thirst (the WUT principle), as the context for changes in body water balance and practical limitations of each method dictate biomarker selection. A framework matching assessment tools to circumstances should be developed to guide clinical and research practice.
Absence of consensus definitions for hydration states
Precise, consensus-backed definitions of hydration states, including euhydration, underhydration, dehydration, overhydration, and hyperhydration, are absent from the scientific literature and are needed to anchor clinical practice, regulatory claim language, consumer education, and future research design.
VII. Microbiome and hydration
Hydration, microbiome, and immune system
Hydration influences the microbiome and the immune system, as shown primarily in animal studies. Reduced water intake can delay gut transit and reduce resistance to enteric infections. The relationship between hydration status and microbial and immune biology in humans remains a mechanistic hypothesis and a priority research opportunity. Clinical evidence is limited, and further human trials are needed.
VIII. Barriers to adequate hydration
Euhydration, barriers, and a population-level challenge
Euhydration can have a positive impact on physical and mental health and exercise performance. Hydration detractors include inconsistency with intake, inconvenience, medication effects, low-calorie and restricted carbohydrate diets, and an imbalance of fluid intake relative to energy output. Achieving adequate hydration at a population level requires a coordinated effort across individuals, health care providers, public health institutions, industry, and policy.

Abbreviation: WUT, body weight, urine color, and thirst.

**TABLE 3:** Actionable items and future research based on information and knowledge gaps

• Formally define the various states of hydration for clarity. Uniform hydration definitions are needed for consistency in clinical settings, regulatory language, consumer education, and future research design.
• Develop guidance on matching specific assessment tools/biomarkers to specific circumstances.
• Strengthen government interest and policy attention to hydration as a public health priority.
• Recategorize beverage groups in line with current market offerings and nutrient database definitions. Confirm that NHANES and national food composition databases fully capture new beverage subcategories (e.g., electrolyte/enhanced waters, flavored waters, and sports/energy drinks).
• Conduct an updated NHANES analysis (2018–2023) on water intake that reflects the most current categories of beverage groups.
• Research on the associations between hydration status, serum sodium, and physiological/disease outcomes (e.g., inflammation and chronic kidney disease).
• Human clinical trials targeting hydration/water biochemical changes, microbiota changes, and metabolic outcomes/health associations.
• Research to determine the contribution of individual electrolytes (e.g., sodium, potassium, calcium, and magnesium) and ratios to osmolality and hydration status.

There are numerous biomarkers of hydration status (Table 1). However, some of these biomarkers appear to be more representative of a particular hydration status than others. Furthermore, hydration status, specifically in response to physical activity, may be best measured using certain biomarkers. For this reason, an important action identified at this meeting was to develop guidance that matches the biomarkers that best represent the hydration status for different situations.

The evidence linking water and sodium intakes with disease outcome, as well as the involvement of vasopressin, is mainly based on animal or observational studies. Neither type of study is sufficient to develop nutrition policies. As such, clinical trials are much needed to establish the relationship between water- and nutrient-induced vasopressin activity and the further causal effect on metabolic and clinical endpoints. Much research has been conducted on the role of electrolytes in hydration and, specifically, on sodium and potassium. Other electrolytes (e.g., magnesium) have a certain level of involvement and are often included in certain beverages (e.g., electrolyte beverages and sports drinks). Research is warranted to further understand the contribution and optimal range of the individual electrolytes (e.g., sodium, potassium, calcium, and magnesium) and specific electrolyte combinations to optimal hydration. Research consistently demonstrates how diet and nutrients influence the colonic microbial population and affect health. The limited evidence on water consumption and effects on colonic bacteria warrants further investigation into water’s role in health.

There needs to be increased government interest in the importance of hydration to Americans. The Dietary Guidelines for Americans are used by various segments of the Federal government to inform nutritional standards, including USDA school lunch programs, food assistance programs to low-income families (e.g., USDA Supplemental Nutrition Assistance Program and USDA Women, Infant, and Children Program), and feeding programs for the elderly (e.g., Meals on Wheels).

## Author contributions

The authors’ responsibilities were as follows – PRT, MT: designed, wrote, and edited the manuscript; LJB, DJC, AD, JAG, RJJ, SK, CXM, MT: critically reviewed and edited the manuscript; and all authors: approved the final content of the manuscript.

## Data availability

Data described in the manuscript, code book, and analytic code will not be made available because there is no data to share for this Perspective article. All information relied upon are based on the references cited in the reference list.

## Funding

This work was supported by Ultima Health Products, Inc. This article, in part, includes information from the Daily Hydration Roundtable held April 8 and 9, 2026, in Bernardsville, NJ, which included presentations and dialogue among experts representing various areas of hydration and health. PRT received funding to collect scientific information, participate in the roundtable, and to prepare the manuscript. MT received funding to develop a scientific strategy and charter for the roundtable, collect scientific information, onboard relevant experts, and chair the roundtable. LJB, DJC, AD, JAG, RJJ, SK, and CXM received travel funding and honorarium to participate in the April 2026 meeting.

## Declaration of generative AI and AI-assisted technologies in the writing process

No AI tool was used in the writing of the manuscript.

## Conflict of interest

MT served as a consultant for Ultima Replenisher, Simply Good Foods, Nestlé, the Hershey’s company, Verley, Blue Diamonds, McCormicks, The National Pork Board, The Almond Board of California, Balchem, Allianza, Chobani, Once upon a Farm, Eden Brew, Mycotechnology, The Protein Brewery, Bactolife, Alt Collective, Alpha Site, Benson Hill, Sound Agriculture, Umiami, Melibio, Bolder, Brain, Noblegen, Calyxt, Center for Food Integrity, Circe Bioscience, Gingko Bioworks, Culinex, Formo, Clareo, Kraft Heinz, Food Strategy Associates, Novo Nordisk foundation, Hoolihan Lockey, Emerald Technology Ventures, LFE Capital, DIC Corporation, Perfeggt, Elo Life, Emerid, Formo, Gosh Foods, Narayan, Eggmented, Reality, More Foods, and John B. Sanfilippo & Son Inc. PRT has served as a consultant or expert witness to KraftHeinz, General Mills, PepsiCo, Johnson & Johnson, Nestlé USA, Ocean Spray, GlaxoSmithKline, Ultima Health Products, Tate & Lyle, Ingredion, Bioneutra, Lantmännen, Hayashibara, MycoTechnology, Quebec Maple Syrup Producers, Colgate Palmolive, Almond Board of California, Constellation Brands, Kappa Biosciences, Kodiak Cakes, Bay State Milling, Intertek, The Protein Brewery, 8Greens, GRAS Associates, ILSI North America, and Institute for the Advancement of Food and Nutrition Sciences. DJC has consulted with and/or conducted research for PepsiCo, Liquid IV, Abbott, Kraft Heinz, and Epicore. AD is or has been a member of scientific advisory panels for BEL, Lesaffre, Nestlé, Friesland Campina, National Pork Board, and Carbohydrate Quality Panel, supported by Potatoes USA. AD has worked with Ajinomoto, Ayanabio, DSM-Firmenich, FoodMinds, KraftHeinz, Meiji, MS-Nutrition, Nutrition Impact LLC, Nutrition Institute, PepsiCo, Samsung, and Soremantec on quantitative ways to assess the nutrient density of foods. JAG serves on the scientific advisory board for Oath Biome, Wonderlabs, Flore, Bened Life, BiomeSense, and Holobiome Inc. CXM is or has been a member of the scientific advisory board for Liquid IV and has served as a consultant for Chobani and KraftHeinz. SK has consulted with and/or conducted research grants for the National Science Foundation, USDA, the American Beverage Association, Kraft Heinz, Unilever, the Coca-Cola Company, Standard Process, Luke Air Force Base, Hyduro Inc, Zico Rising, Trace Minerals, Rockley Photonics, Liquid IV, Unilever, and PepsiCo. He is also the owner of Dr. Hydration LLC.
